# Glasgow Royal Infirmary
*Glasgow Medical Journal, No. XI


**Published:** 1831-01-01

**Authors:** 


### GLASGOW ROYAL INFIRMARY.*

### RETENTION OF URINE.

WE scarcely know a more important subject to the surgeon than retention of urine and its consequences. The mortality in hospitals from cases of old stricture and urinary abscess is considerable; and although the principles of treatment may be ascertained, the

* Glasgow Medical Journal, No. XI.

practice is too often unsuccessful. Mr. Cowan, one of the surgeons to the Glasgow Infirmary, has published some cases of retention of urine in a report of the cases under his care from November 1, 1829, to May 1, 1830.

*Case* 1. M. D. æt. 60, admitted with great distention of the bladder, stillicidium urinæ to some extent, pain in the back and loins, faintness, and anxious countenance. He had experienced some difficulty in making water for six months, but since intoxication five days previously had been unable to void it. For two days it had been drawn off, but during the last three the surgeon had failed in introducing the catheter.

A full sized catheter was passed into the bladder, and ℔iij. of urine evacuated. By the warm bath and introduction of the catheter thrice daily, the bladder regained its tone and the patient was dismissed. The middle lobe of the prostate was enlarged and had occasioned the obstruction.

*Case* 3. J. M., ætatis 30, admitted February 15. Had gonorrhœa four years ago—retention of urine, removed by the catheter, six months ago, and since that, difficulty of making water—complete retention after intoxication two days ago. On attempting to pass a catheter blood only has been discharged. Has at present retention, pain above the pubes, and stillicidium urinæ. An instrument passes as far as the bulb, but beyond this it enters a false passage and occasions hæmorrhage.

A bougie was introduced as far as the stricture, and kept firmly in contact with it, and a purgative clyster administered. He was afterwards placed in the warm bath, and took ten drops of the tincture of tobacco every hour. In some hours he had passed a considerable quantity of urine, and by the visit on the 18th, he was completely relieved. A full-sized bougie was ordered to be passed down to the stricture thrice daily, and kept for some time firmly applied to it. By persevering in this plan, the stricture was overcome, and a full-sized silver sound having been for a length of time passed into the bladder, he was dismissed cured on the 8th March.

*Case* 4. M. D. æt. 56, of broken constitution, admitted Dec. 1, 1829, with prepuce and scrotum much swollen; hard, painful swelling in right groin and round root of penis; incessant desire to make water and involuntary discharge of it in some quantity; great distention of the bladder. The patient has much tremor, pulse 120, tongue brown, diarrhœa and epistaxis. Has had stricture for sixteen years, a rigor six days ago. The catheter, about three inches from the orifice of the urethra, passed into the tumour at the root of the penis, from which urinous pus was discharged. About an inch anterior to the anus, the catheter was stopped by a small hard tubercle distinctly felt in perineo. Here an incision was made down upon the end of the instrument, a full sized catheter introduced, and the tumour divided. The membranous portion of the urethra behind it was then opened, and a female catheter introduced into the bladder, when a great quantity of very fœtid urine was drawn off with immediate relief. The abscess at the root of the penis was laid open, and the penis and scrotum freely scarified. The patient died on the 26th.

On dissection, the abscess was chiefly on the right side, and extended over the abdominal parietes nearly to the umbilicus. Its parietes were sloughy; the opening into it from the superior surface of the urethra ragged and sloughy also, the corpora cavernosa penis destroyed, and no remains of stricture to be discovered. The bladder was not contracted, nor the prostate gland enlarged.

In the first of these cases, in which the prostate gland was enlarged, there can be little doubt that the failure in the hands of the first surgeon arose from employing too small or too short an instrument. In cases of this kind the instrument should always be full-sized, long, and, as Mr. Hey has observed, tolerably curved at the extremity. Indeed, as a general rule, we should employ a large instrument in preference to a small one in all cases whatever.

In the second case, as the stream of urine was, before the retention took place, as large nearly as a crow-quill, it was conceived that the retention depended partly on a spasmodic stricture, superadded to the mechanical obstruction. As the retention had existed for two days, the necessity for relieving the bladder was great; but on introducing the catheter, it was discovered, what indeed was inferred from the repeated hæmorrhage before admission, that the urethra had been lacerated by the attempts at introduction, and that one or two false passages existed. It was therefore deemed prudent not to continue the attempts at catheterism, but to trust to other means, which had been found successful in other instances.

When a catheter is carried down to a stricture, and kept in strict contact with it, a discharge from the urethra takes place, and when this has been aided by the warm bath, purgatives, and the use of tobacco tincture, in almost every case a cure will be effected. In cases similar to the present, the practice has invariably succeeded with me, and is to be preferred to repeated attempts to pass the catheter, where laceration of the urethra has been produced by former efforts.

Mr. Cowan thinks the third case of urinous abscess remarkable for its situation, but we have several times seen it extending over the pubes to the parietes of the abdomen. Mr. Cowan has operated twice in retention of urine, and each time he has opened the urethra, and passed from the incision a small female catheter into the bladder. Sir Astley Cooper, as our readers are aware, maintains that puncturing the bladder for stricture of the urethra or enlargement of the prostate is unnecessary and unwarrantable. We have witnessed, however, the dissection of cases of old stricture, in which it would have been utterly impossible to have opened the urethra behind the obstruction, in consequence of the inextricable confusion occasioned by false passages and inflammation.

### II. NEURALGIA AND TUMOURS OF NERVES.

After amputation and after injuries the nerves sometimes swell and form small tubercles, which at times are acutely painful, especially on changes of weather. But slight or severe operations in very irritable persons, and particularly in young females, will give rise to painful affections, which appear to depend more on a hysterical or nervous habit, than on organic changes in the nerves. Thus the painful affections in the arms of young women, so frequent after venesection, are in nine cases out of ten altogether unconnected with wound of the nerve, though the contrary is vulgarly imagined to be the case. The following would seem to be an instance of this kind.

*Case* 1. Mr. Cowan amputated the fore-arm of a girl, twenty years of age, at the secondary period, for an extensive wound by machinery. The stump healed, but she then became affected with excruciating attacks of pain in it, extending up the arm, generally commencing at 6 o'clock in the morning and evening, and lasting for some hours. The pain was excited by pressure on the stump at two particular points. She was cured by quinine in full doses, and a relapse shortly afterwards was likewise treated with success in the same manner.

Now it is not improbable that, had this young woman never undergone an operation at all, she would have suffered from some other form of those hysterical pains which are the heritage of her sex. But it is certain that injuries, however inflicted, give a greater disposition to these affections, and determine the part which the malady more especially attacks. In almost all the cases which we have witnessed of hysterical pain in the knee or breast, the patient has referred the origin of her complaint to a blow, real or imagined. The next case is of another description.

*Case* 2. J. R., æt. 36, admitted Jan. 19, 1830, with a small tumour the size of a bean, a little above the left superciliary ridge of the frontal bone towards its inner angle. It is freely moveable, and the seat of severe throbbing pain, extending across the forehead and over the scalp, excited by stooping, coming on in a paroxysm at nine o'clock in the evening and continuing till day-break. The pain prevents sleep. He first observed the tumour when about the size of a pea some years ago, and attributes it to wearing the Scots Greys' helmet. He has had the paroxysms regularly every night for these last twelve months, but latterly they have much increased in severity. Mr. Cowan gave a trial to quinine, but it failed, and on the 23d he removed the tumour by the knife; it was firm in its texture, and appeared to be an enlargement of the nerve, but of this Mr. C. speaks with diffidence. The patient was dismissed cured on the 26th, and remains well.

*Case* 3. J. R. æt. 22, admitted Nov. 28th, with a large, hard, moveable tumour, occupying the posterior part of the lower third of the left thigh, and the upper part of the popliteal space. It presses out the ham-strings laterally, but is unattached to the integuments, which merely display a few enlarged veins. The tumour is devoid of pain, except when pressed laterally or downwards upon the femur, when acute pain is felt, succeeded in a few seconds by an aching on each side, and on the under surface of the heel, from which at intervals darting pains proceed to the toes and dorsum of the foot. These latter are felt independent of pressure on the tumour. The pains are so severe at night that he constantly maintains the erect position, and his sleep is broken by troubled dreams. He has lost flesh, and the appetite is bad. The tumour was first noticed five years ago, the severe pain in the foot commenced nine weeks ago.

During his continuance in the hospital various means were tried without relief. Opium, iodine, leeches, blisters, quinine, were all unsuccessful. The extirpation of the tumour was "out of the question," the patient would not submit to amputation, and on the 27th of January he left the house. He returned in the course of a month, the disease having increased in severity, and about the middle of May the limb was amputated. Mr. Cowan examined the tumour after it was placed in spirits; it was formed by a dilatation of the structure of the tibial nerve which formed the external covering, but internally it seemed wholly to consist of fibrine, separable with ease from its nervous sheath to which it was unattached. The patient died three weeks after the operation with symptoms of affection of the chest.

Mr. Cowan observes that this was an example of the disease *neuroma*, a term first applied by Odier, of Geneva, to tumours formed by diseased enlargements of nerves. A paper on the subject, by Mr. Wood, president of the College of Surgeons of Edinburgh, will be found in Transactions of the Medico-Chirurgical Society of Edinburgh.* We have seen one case of tetanus and heard of another, in which wound of a nerve produced a kind of knot or ganglion upon it, that appeared to be the cause of the tetanic symptoms. But this is a different kind of case from that related by Mr. Cowan.

### III. HYDRO RACHITIS.

*Case.* J. B. æt. 18 months, admitted Jan. 7th, 1830, with a pendulous tumour the size of a hen's egg, between the base of the sacrum and posterior superior spinous process of the ilium, and attached by a firm round pedicle, about three inches in diameter. The tumour is covered with skin unnaturally thick, particularly at its root, and is ulcerated on its surface from the friction of the clothes; it is painful on pressure and devoid of fluctuation; no irregularity of the spinous processes; fontanelles closed; child plump and healthy, and retaining full command of its lower extremities. When born, the tumour was of the size of a hazel-nut, and of bluish colour; it has latterly increased.

Doubts were entertained respecting

---

* Vol. III. Part II.

the nature of the case, particularly whether it was or was not a case of spina bifida. On the 11th, it was punctured and some serous fluid discharged, after which the large fleshy portion of the tumour was removed by the knife. Three arteries of magnitude required to be tied, and in a minute or two a discharge of serum was observed from a small opening, in structure resembling a vein. This opening communicated with the spinal canal. The lips of the wound were brought together with plaster, and compress and bandage applied. The tumour weighed three-fourths of an ounce, was composed externally of preternaturally thickened skin and cellular membrane, and internally of two distinct very small cysts, communicating through a small opening with each other, and lined with a thin smooth highly polished membrane.

Serum in considerable quantity continued to be discharged, and on the 13th the patient was seized with retching and vomiting, and anxiety of countenance. Mr. Cowan, the surgeon, attempted to secure the spinal opening with a ligature, but failed. The pressure was then carefully renewed, two leeches and a blister were applied to the spine, and on the next day the child was greatly better. A small elastic pad was constructed, and the discharge of fluid nearly checked, the little patient improved in appearance, and the wound began to cicatrize. But the mother grew tired of the hospital and removed the child altogether.

"Many circumstances combined to render the diagnosis in this case obscure. Most children with this disease die within one year of their birth, and Ruysch, whose experience was ample, met with one only who attained the age of fifteen months. The age of the above child was eighteen months, his health had been good, as evinced by his general appearance, and in particular by the complete ossification of his skull. No paralytic symptoms had ever existed, and with the exception of the increasing size of the tumour, and its ulceration, little inconvenience had arisen from it. The size of the aperture communicating with the spinal canal was small, and instead, as in many instances, of there being a deficiency of skin over the tumour, whose covering is sometimes merely that derived from the dura mater of the spinal sheath, the integuments of the part were thickened, amply supplied with blood, and formed, independently of the original disease, a swelling of considerable magnitude, so much so indeed, as to render obscure, not only at the consultation, but even after evacuating the contents of the tumour, the nature of the disease.

The error in diagnosis led to the operation, for had the real nature of the complaint been apparent, nothing would have been done except taking such measures as might be requisite for arresting the ulceration of the surface of the tumour, which sooner or later must have penetrated the sac.

The tumour was remarkable too, for being composed of two distinct cysts communicating with each other."

